# AMPK inhibitor, compound C, inhibits coronavirus replication *in vitro*

**DOI:** 10.1371/journal.pone.0292309

**Published:** 2023-10-03

**Authors:** Minsu Jang, Rackhyun Park, Ayane Yamamoto, Yea-In Park, Yeonjeong Park, Siyun Lee, Junsoo Park

**Affiliations:** 1 Division of Biological Science and Technology, Yonsei University, Wonju, Republic of Korea; 2 Department of Life Science, Yong-In University, Yongin, Republic of Korea; Sathyabama Institute of Science and Technology, INDIA

## Abstract

The coronavirus disease (COVID-19) pandemic has resulted in more than six million deaths by October 2022. Vaccines and antivirals for severe acute respiratory syndrome coronavirus 2 are now available; however, more effective antiviral drugs are required for effective treatment. Here, we report that a potent AMP-activated protein kinase (AMPK) inhibitor, compound C/dorsomorphin, inhibits the replication of the human coronavirus OC43 strain (HCoV-OC43). We examined HCoV-OC43 replication in control and AMPK-knockout (KO) cells and found that the virus replication decreased in AMPK-KO cells. Next, we examined the effect of the AMPK inhibitor, compound C on coronavirus replication. Compound C treatment efficiently inhibited the replication and decreased the coronavirus-induced cytotoxicity, further inhibiting autophagy. In addition, treatment with compound C in combination with chloroquine synergistically inhibited coronavirus replication. These results suggest that compound C can be considered as a potential drug candidate for COVID-19.

## Introduction

The coronavirus disease (COVID-19) pandemic has resulted in millions of deaths owing to the high infectivity and high mortality of severe acute respiratory syndrome coronavirus 2 (SARS-CoV-2) [1]. Although vaccines for SARS-CoV-2 are currently available, effective drugs for COVID-19 are required. Therefore, many researchers have focused on the development of effective treatment for coronavirus diseases using various cell signaling pathways, which are potentially important against coronavirus replication.

Macroautophagy (autophagy) is an evolutionarily conserved catabolic process that requires lysosomal function, however, its involvement in coronavirus replication is controversial [2, 3]. Coronavirus infection results in the formation of double membrane structures containing autophagy proteins, including LC3 and ATG12. Mouse hepatitis virus replication is impaired in ATG5-knockout (KO) cells [4, 5]. However, several reports also support that autophagy is not critical for coronavirus replication, as the deletion of key autophagy regulators did not affect coronavirus replication [6–8]. While the role of autophagy in coronavirus replication is unclear, the autophagic flux inhibitors chloroquine and hydroxychloroquine have been reported to efficiently inhibit coronavirus replication [9, 10]. They interfere with the replication of SARS-CoV and SARS-CoV-2 *in vitro* by elevating vesicular pH and regulating the glycosylation of angiotensin converting enzyme 2 [9, 11, 12]. However, clinical trials have not provided consistent and convincing evidence, with studies demonstrating no benefits for the use of chloroquine and hydroxychloroquine in COVID-19 patients [13, 14].

Compound C, also known as dorsomorphin, is a potent AMP-activated protein kinase (AMPK) inhibitor widely used to block AMPK-dependent signaling [15]. Compound C is shown to induce AMPK-independent autophagy by subsequently inhibiting Akt and mTOR [16, 17]. In addition to AMPK inhibition, compound C inhibits bone morphogenetic protein (BMP) and VEGF type 2 receptor signaling [18, 19] and has anti-proliferative effects on cancer cells, including those in colorectal cancer, skin cancer, and glioma [17, 20, 21].

Coronaviruses are classified into alpha, beta, gamma, and delta coronaviruses, where alpha and beta coronaviruses can infect humans. So far, seven coronaviruses have been known to infect human, including human coronavirus 229E (HCoV-229), HCoV-HKU1, HCoV-NL63, HCoV-OC43, SARS-CoV, MERS-CoV and SARS-CoV-2 [22]. SARS-CoV-2 and HCoV-OC43 belong to the beta-coronavirus family [23]. Owing to the strict conditions for dealing with SARS-CoV-2, we used the HCoV-OC43 virus as a surrogate in this report. We aimed to assess the OC43 coronavirus infection in AMPK-KO cells. Further, we used an AMPK inhibitor, compound C, to study coronavirus replication and coronavirus-induced cytotoxicity. We believe that our findings will aid in identifying a novel drug target for coronavirus diseases.

## Materials and methods

### Cell culture and infection

The HCoV-OC43 virus was obtained from ATCC (Rockville, MD, USA), and Rhabdomyosarcoma (RD) cells were obtained from the Korean Cell Line Bank (KCLB, Seoul, Korea). RD cells were maintained in MEM (Welgene, Seoul, Korea) containing 10% fetal bovine serum (FBS, Thermo Fisher Scientific, Waltham, MA, USA) and 1% penicillin-streptomycin (Welgene). RD cells were infected with HCoV-OC43 as described previously [24]. Cell viability was measured using the 3-(4,5-dimethylthiazol-2-yl)-2,5-diphenyltetrazolium bromide (MTT) assay [25]. MTT was purchased from USB Corporation (Cleveland, OH, USA). Cells were treated using compound C (Sigma-Aldrich, Saint Louis, MO, USA).

### Plaque formation assay

To enumerate the virus, RD cells were seeded in a 12-well plate one day prior to the assay. The viral samples were diluted 10-fold in MEM with 2% FBS and added to the corresponding wells and incubated for 1 h at 33°C and 5% CO_2_. Overlay medium was prepared as a mixture of 0.6% agarose in MEM, of which 1 mL was added to each well and the plate was incubated for 5 days. Finally, the cells were fixed with a 10% formaldehyde solution and stained with 1% crystal violet. HCoV-OC43 virus titer in the media was 10^7^ PFU/ml.

### Quantitative reverse transcription polymerase chain reaction (RT-PCR)

Quantitative RT-PCR was used to measure the level of coronavirus RNA in cells and media, as previously described [24]. Briefly, cells and media were harvested, and RNA was extracted using Trizol (Thermo Fisher Scientific) in accordance with the manufacturer’s instructions and then subjected to RT-PCR using the StepOnePlus Real-Time PCR System (Thermo Fisher Scientific). HCoV-OC43 RdRp mRNA was amplified using the forward primer 5′- GATGTAGATGCCCGTCTCG -3′ and reverse primer 5′- TGTGGCACACGACTACCTTC -3′. HCoV-OC43 M mRNA was amplified using the forward primer 5′- ACGGTCACAATA ATACGCGGT -3′ and reverse primer 5′- GGGTTGATGGCAGTCGGTAA -3′. The HCoV-OC43 N mRNA was amplified using the forward primer 5′- AGGATGCCACCAAACCTCAG -3′ and reverse primer 5′- TGGGGAACTGTGGGTCACTA -3′. The input RNA was normalized via the amplification of ribosomal protein L4 (RPL4) RNA with the forward primer 5′-GCTCTGGCCAGGGTGCTTTTG-3′ and reverse primer 5′-ATGGCGTATCGTTTTTGGGTTGT-3′.

### Western blotting

Western blotting was used to measure the level of coronavirus protein in cells and media, as described previously [24, 26]. Briefly, cells and media were collected and resuspended in cell lysis buffer (150 mM NaCl, 50 mM HEPES (pH 7.5), and 1% NP40) containing a protease inhibitor cocktail (Roche, Basel, Switzerland). Cell lysates were resolved by SDS-PAGE and transferred to immune-blot PVDF membrane filters (Bio-Rad, Hercules, CA, USA). Viral proteins were detected with a 1:5000 dilution of primary HCoV-OC43 antibody using an ECL Western blotting substrate (Dogen, Seoul, Korea). The images were acquired using the ChemiDoc Imaging System (Bio-Rad). The HCoV-OC43 antibody was purchased from Sigma-Aldrich.

### Immunofluorescence analysis

To visualize the cells infected with coronavirus, cells were grown on sterilized glass coverslips and infected with coronavirus. The cells were fixed with 4% paraformaldehyde, blocked with 3% bovine serum albumin in PBS, and stained with anti-OC43 antibody (1:1000 dilution). Finally, the cells were stained with Alexa Fluor 488–conjugated secondary antibody (Thermo Fisher Scientific) and DAPI. Images were captured using a Carl Zeiss LSM710 confocal microscope (Carl Zeiss, Oberkochen, Germany). Both OC43 positive cells and OC43 negative cells were counted and analyzed to show the percentage of HCoV-OC43 infected cells in each microscopy image.

### Statistical analysi

The results of western blotting, quantitative RT-PCR, and MTT assay were evaluated by a two-tailed Student’s t-test using Excel software (Microsoft, Redmond, WA, USA). Statistical significance was set as *P* < 0.05.

## Results

### Coronavirus replication is decreased in AMPK-KO cells

**Fig 1:**
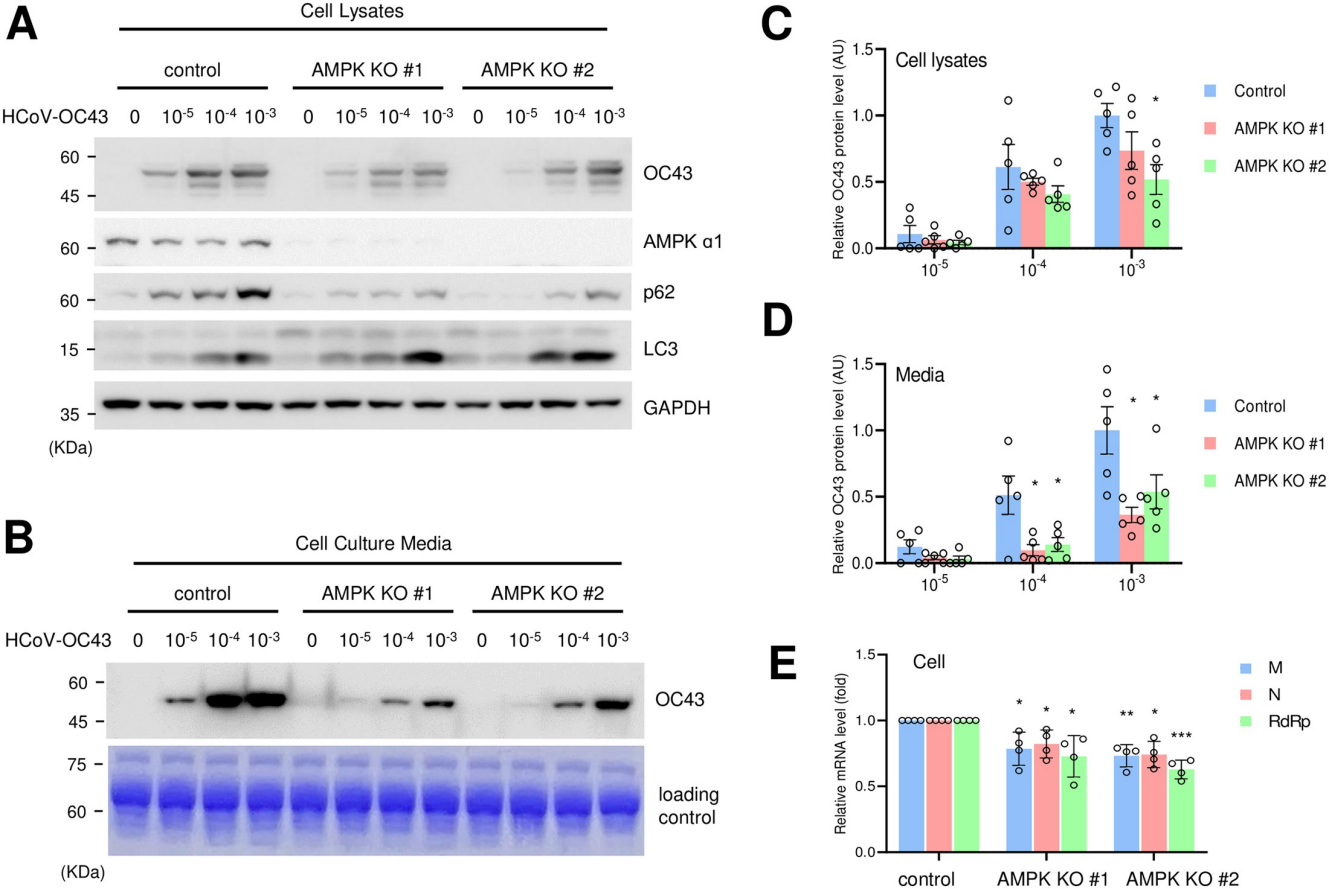
Coronavirus replication is decreased in AMPK-knockout (KO) cells. (A) The expression level of coronavirus proteins in AMPK-KO cells. HEK293T control cells and AMPK-KO cells were infected with the indicated dilutions of HCoV-OC43 coronavirus. Cells were collected after 72 h of infection and equal amount of cell lysates were subjected to western blotting with the indicated antibodies. (B) The expression level of coronavirus proteins in the media of AMPK-KO cells. Media of virus-infected cells were collected and probed with anti-HCoV-OC43 antibody. Coomassie staining of media was used as a loading control. (C and D) The level of OC43 proteins were quantitated and denoted as a graph in cell lysates (C) showing mean ± standard error and in media (D) showing mean ± standard deviation. Control vs KO cells: *, *P* < 0.05, n = 5. (E) HEK293T control and AMPK-KO cells were infected with coronavirus (10^−3^ dilution), and RNA was collected from the media. The RNA level of RdRp gene, M gene and N gene was evaluated using quantitative RT-PCR. Control vs KO cells: *, *P* < 0.05, **, *P* < 0.01, ***, *P* < 0.005, n = 4.

Several studies have suggested that autophagy is involved in coronavirus replication; Thus, we attempted to examine the role of AMPK in coronavirus replication. Previously, we generated AMPK-KO cells and showed that autophagic flux is deregulated in these cells [27]. Here, control and AMPK-KO cells were infected with HCoV-OC43 coronavirus, and western blotting with anti-OC43 antibody was used to examine the level of coronavirus. The level of coronavirus in the cells was significantly decreased in AMPK-KO cells (Fig 1A and 1C). Moreover, coronavirus in the cell culture media was also dramatically decreased in AMPK-KO cells (Fig 1B and 1D). Further, we examined the level of coronavirus RNA in the cell culture media using quantitative RT-PCR and found a significant reduction in AMPK-KO cells (Fig 1E). These results indicate that AMPK is required for efficient coronavirus replication.

To assess whether AMPK activity and coronavirus infection contribute to autophagy regulation, we examined the level of autophagy marker expression. Coronavirus infection increased the level of LC3-II protein and p62 (Fig 1A), which indicated that coronavirus infection affected autophagy. We also found that AMPK-KO cells showed a slightly lower level of p62 protein following coronavirus infection (Fig 1A).

### Compound C treatment inhibits coronavirus replication

**Fig 2:**
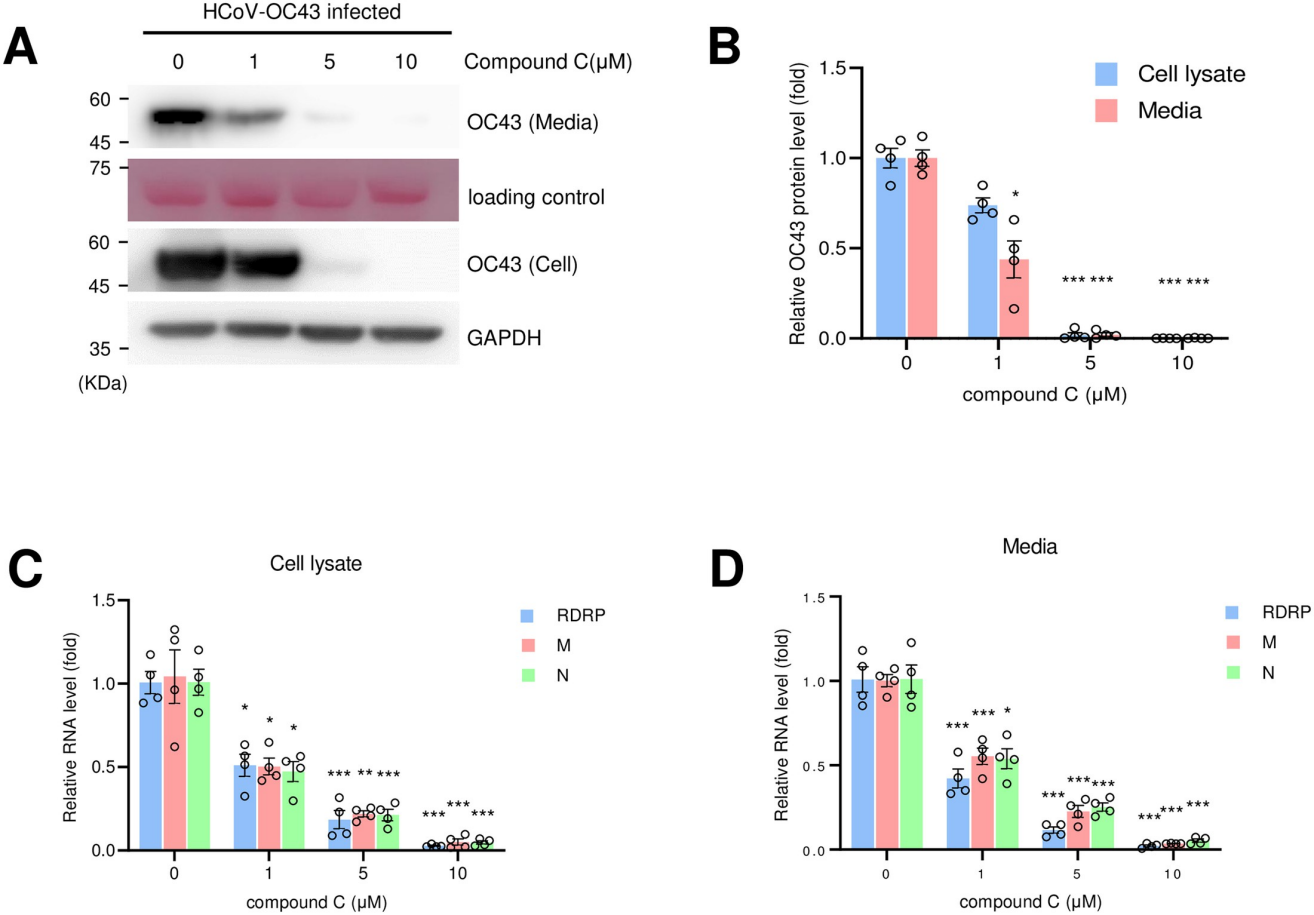
Compound C treatment inhibits coronavirus replication. (A) RD cells were infected with coronavirus (10^−3^ dilution) and incubated with the indicated concentrations of compound C. Cells and media were collected after 72 h of infection and probed with anti-HCoV-OC43 antibody. Ponceau S staining was used as a loading control for media western blot. (B) The expression of coronavirus protein was q uantitated and denoted as a graph. Control vs compound C treated: *, *P* < 0.05, ***, *P* < 0.005, n = 4. (C and D) RD cells were infected with coronavirus, and incubated with the indicated concentration of compound C. After 72 h of infection, RNA was collected from the cells (C) and media (D) and the RNA level of RdRp gene, M gene and N gene was evaluated by quantitative RT-PCR. Control vs compound C treated: *, *P* < 0.05, **, *P* < 0.01, ***, *P* < 0.005, n = 4.

The replication of coronavirus was decreased in AMPK-KO cells; therefore, we hypothesized that AMPK inhibition by AMPK inhibitor would also decrease the replication. As predicted, AMPK inhibitor, compound C treatment decreased the level of coronavirus protein in the cell culture media and cell lysates in a dose-dependent manner (Fig 2A and 2B). We further examined the level of coronavirus RNA, which also decreased after compound C treatment in a dose-dependent manner in both cells and culture media (Fig 2C and 2D). These results indicate that the inhibition of AMPK by compound C affected coronavirus replication.

### Compound C treatment decreases coronavirus cytotoxicity

**Fig 3:**
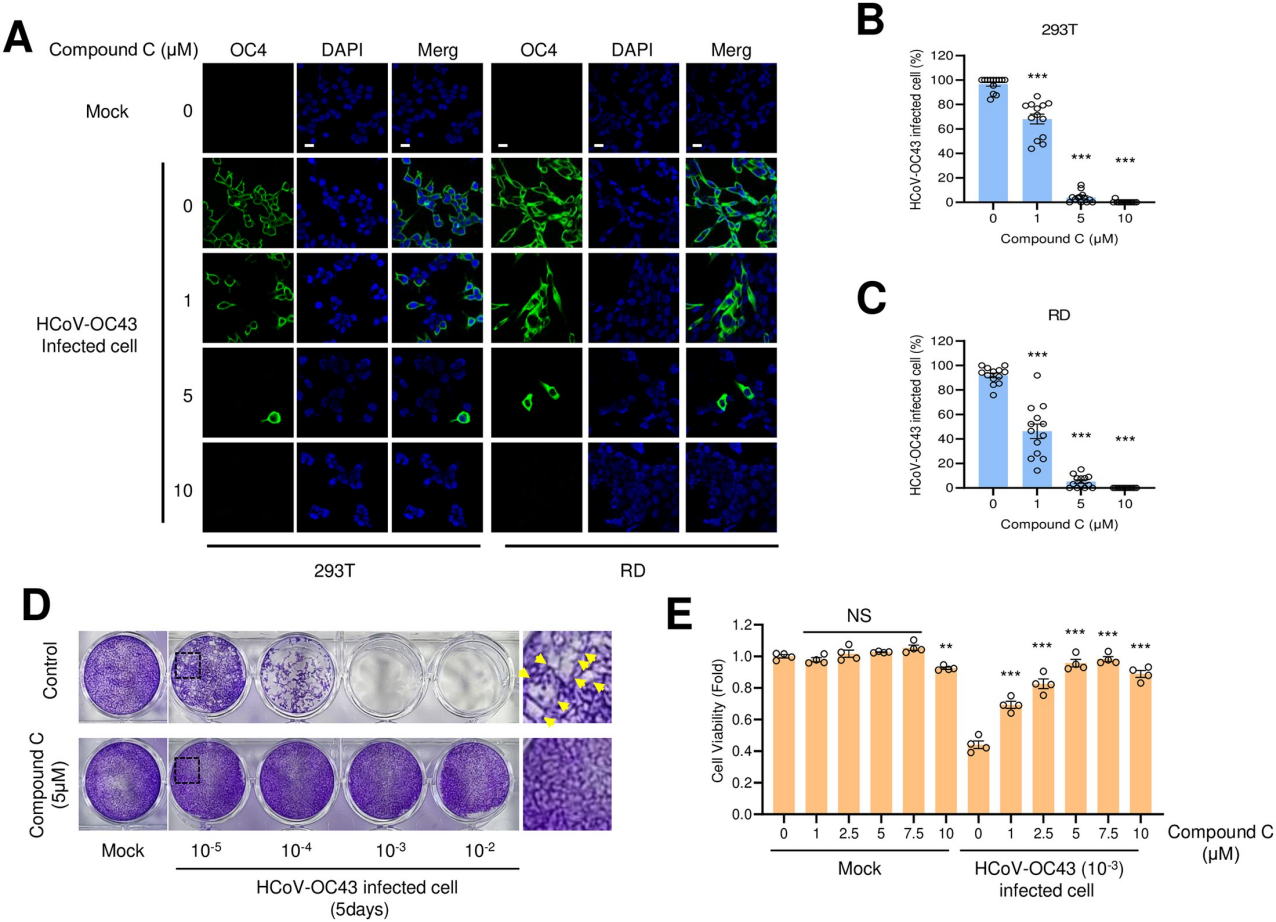
Compound C treatment inhibits the coronavirus infection. (A) HEK293T cells and RD cells were incubated with coronavirus (10^−3^ dilution), and with the indicated concentrations of compound C. Cells were fixed 48 h after infection and stained with anti-HCoV-OC43 antibody. Bars: 10 μM. (B and C) Coronavirus infected cells were counted and the percentage of infection was calculated in HEK293T cells (B) and RD cells (C). Control vs compound C treated: ***, *P* < 0.005, n = 13. (D) Compound C treatment induced HCoV-OC43 cytotoxicity. RD cells were infected with mock or HCoV-OC43 and treated with control or compound C followed by agarose overlay. To visualize the cytotoxicity, the infected cells were fixed and stained with crystal violet. (E) RD cells were infected with mock or HCoV-OC43 and incubated with control or compound C for 5 days. Cell viability was measured by MTT assay. ***, *P* < 0.005, n = 4.

We further examined the effects of compound C treatment on coronavirus infection using immunofluorescence staining. HEK293T and RD cells were infected with HCoV-OC43 virus and incubated with compound C, and viral protein expression was assessed using immunofluorescence staining with anti-OC43 antibody. With an increase in the compound C concentration, the number of coronavirus-positive cells decreased significantly, indicating that compound C treatment inhibited coronavirus infections (Fig 3A–3C).

Since compound C treatment interferes with coronavirus replication, we expected that the cytotoxicity caused by the infection would be decreased by compound C treatment. In a plaque formation assay, while coronavirus infection induced plaque formation, compound C treatment reduced it (Fig 3D). This suggests that compound C decreases the cytotoxicity of coronavirus.

Furthermore, examination of cell viability upon coronavirus infection in the presence or absence of compound C showed a significant decrease in cell viability post-infection, which was alleviated with compound C treatment (Fig 3E). These results indicate that compound C protects cells from the coronavirus-induced cell death.

### Compound C and chloroquine treatments synergistically inhibits coronavirus replication

**Fig 4:**
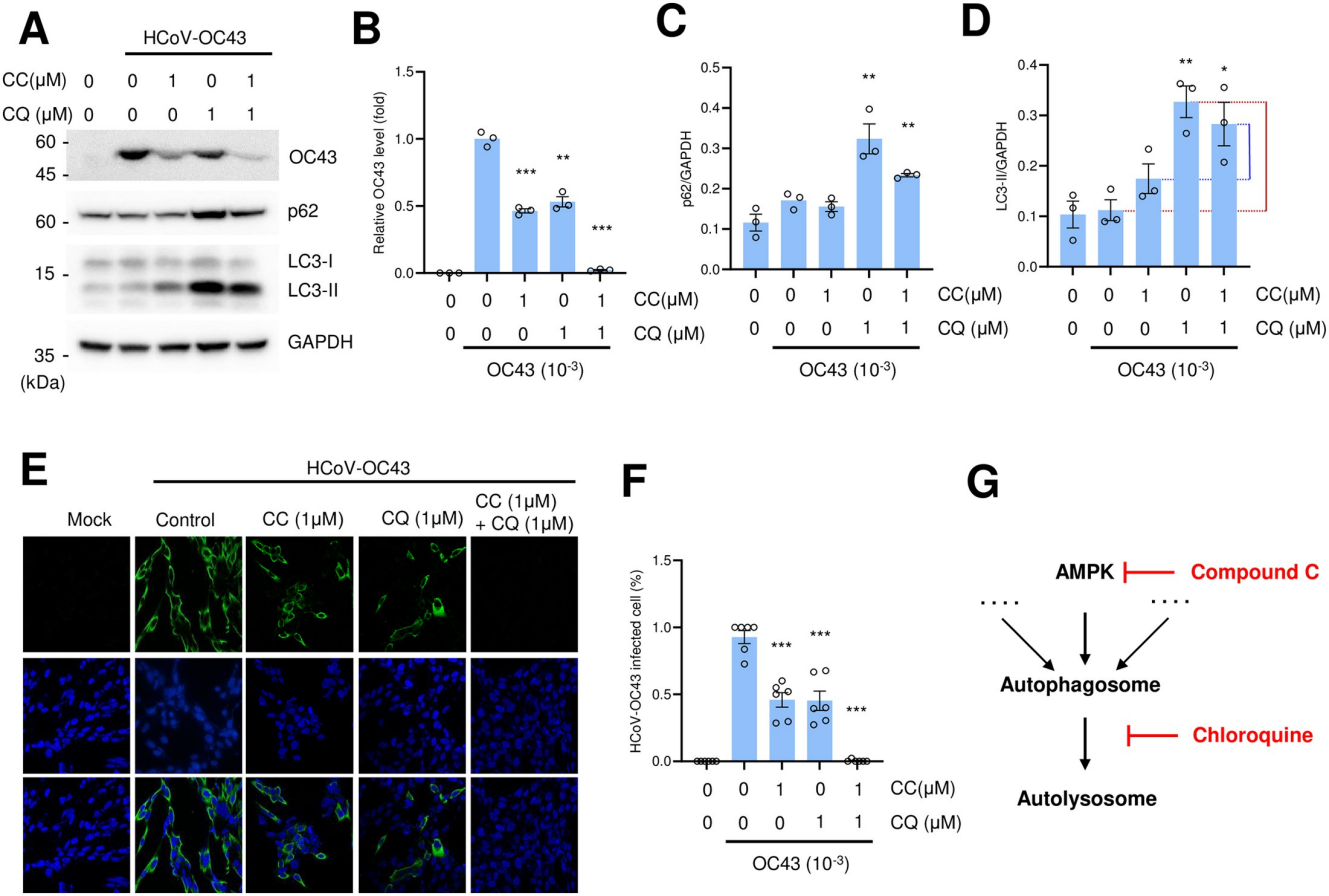
Combination of compound C (CC) and chloroquine (CQ) treatment synergistically inhibits the coronavirus infection. (A) HEK293T cells were incubated with coronavirus (10^−3^ dilution), and incubated with the indicated concentrations of CC and CQ. Cells were collected 48 h after infection and subject to western blot with anti-HCoV-OC43 antibody. (B) The level of OC43 proteins were quantitated and denoted as a graph. **, *P* < 0.01, ***, *P* < 0.005, n = 3. (C and D) The expression levels of p62 and LC3 were quantitated and denoted as a graph. **, *P* < 0.01, n = 3. (E) HEK293T cells were incubated with coronavirus (10^−3^ dilution), and incubated with the indicated combinations of CC and CQ. Cells were fixed 48 h after infection and stained with anti-HCoV-OC43 antibody. Bars, 10 μM. (F) Coronavirus infected cells were calculated in HEK293T cells and denote in graph. ***, *P* < 0.005, n = 6. (G) Schematic diagram showing the action of compound C and chloroquine on the autophagy.

As AMPK inhibition interferes with autophagy, we hypothesized that an autophagy and AMPK inhibition may synergistically inhibit coronavirus replication. Toward this, we treated cells with chloroquine, a representative inhibitor of autolysosome formation, and compound C (Fig 4G). While compound C and chloroquine inhibited the expression of coronavirus protein individually, the co-treatment showed a synergistic effect (Fig 4A and 4B). Additionally, immunofluorescence staining revealed that co-treatment with compound C and chloroquine synergistically decreased the percentage of coronavirus-infected cells (Fig 4E and 4F).

We also examined the expression of autophagy markers in combination with compound C and chloroquine treatment following coronavirus infection (Fig 4C and 4D). We measured autophagic flux based on differences in LC3-II protein expression in the presence or absence of chloroquine. The autophagic flux, as determined by the LC3-II protein levels was also decreased in the presence of compound C (Fig 4D). The graph indicates that compound C treatment reduced the autophagic flux (Fig 4D).

## Discussion

In this study, we examined the effect of compound C/dorsomorphin on the replication of human coronaviruses. Previously, we generated AMPKɑ1-KO cell lines and demonstrated that autophagy initiation and progression were impaired [27]. Here, we used AMPK-KO cells to examine the role of AMPK in coronavirus replication and found it to be an essential factor. We used AMPK-KO cell data to develop potential coronavirus drugs. Next, we tested an AMPK inhibitor, compound C and found that it effectively inhibited coronavirus replication. These results suggest that AMPK plays an important role in coronavirus replication and is a novel drug target for coronavirus disease.

Compound C induces apoptosis in cancer cells [17], and we also observed slight cytotoxicity at higher concentrations (≥10 μM). In cell culture experiments, we found that up to 7.5 μM did not show significant cytotoxicity and significantly decreased coronavirus-induced cytotoxicity (Fig 3E). Although the toxicity of compound C has not been fully examined in animals, recent studies have shown that up to 15 mg/kg of compound C can be injected into mouse [28]. These concentrations can inhibit coronavirus replication; therefore, compound C can potentially be useful for treating coronavirus disease *in vivo*. Although compound C is reported to show an inhibitory effect on several kinases other than AMPK, currently, no AMPK-specific inhibitor is available, and the development of more specific inhibitors would be helpful in treating coronavirus-related diseases [29].

In addition, we demonstrated that co-treatment with compound C and chloroquine collectively inhibited coronavirus replication (Fig 4). The dose of compound C and chloroquine can be further decreased during the administration of both the drugs, where the cytotoxicity can also be reduced. Further studies, including animal experiments, should be performed to validate the efficacy of compound C and chloroquine *in vivo*.

In this report, we demonstrated that AMPK-KO and an AMPK inhibitor downregulated coronavirus replication. Recent reports have highlighted the requirement of autophagy for efficient replication of coronavirus and that autophagic flux can be blocked by coronavirus protein expression [2, 30]. Interestingly, we showed that coronavirus infection induced the expression of p62 indicating that autophagic flux was suppressed (Fig 1A). Co-treatment with compound C and chloroquine efficiently blocked coronavirus replication where compound C can inhibit autophagy initiation, and chloroquine treatment interferes with autophagic flux. This co-treatment efficiently reduced autophagy (Fig 4G). Moreover, these findings suggest that autophagy inhibition contributes to the reduction in coronavirus replication. Both the drugs affected different pathways, therefore, there may be additional signaling pathways that regulate coronavirus replication, which needs to be further elucidated.

HCoV-OC43, which belongs to the beta coronavirus family like SARS-CoV-2, induces a mild disease [22]. Therefore, HCoV-OC43 has been widely used to study the coronavirus [24]. Here, we used HCoV-OC43 as a model system to study SARS-CoV-2, and further study is required to examine and validate the effect of compound C on SARS-CoV-2 replication. Moreover, the current study was performed *in vitro*, and animal experiments should be performed to validate this effect *in vivo*.

## Supporting information

S1 Raw images (PDF)
