# Supplementary figures and images for: AMPK inhibitor, compound C, inhibits coronavirus replication in vitro
Source: PLoS One. 2023 Oct 3;18(10):e0292309. doi: 10.1371/journal.pone.0292309 (PMC10547180; doi:10.1371/journal.pone.0292309)

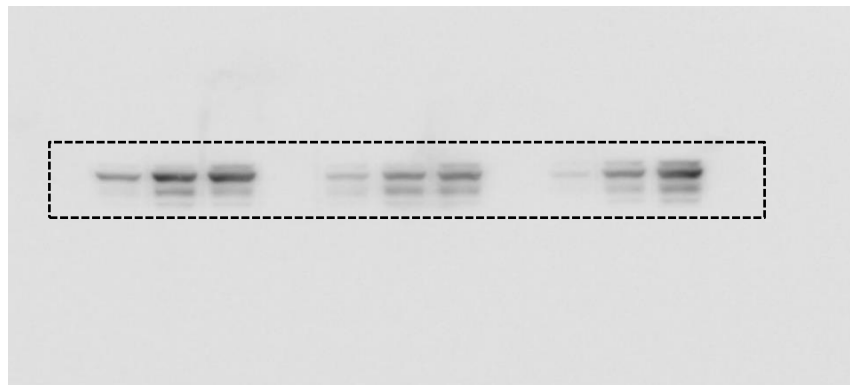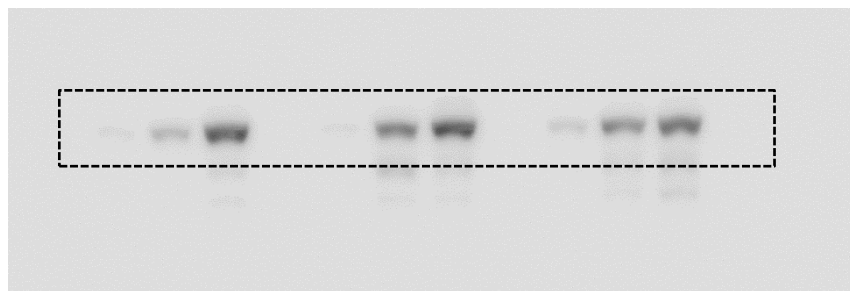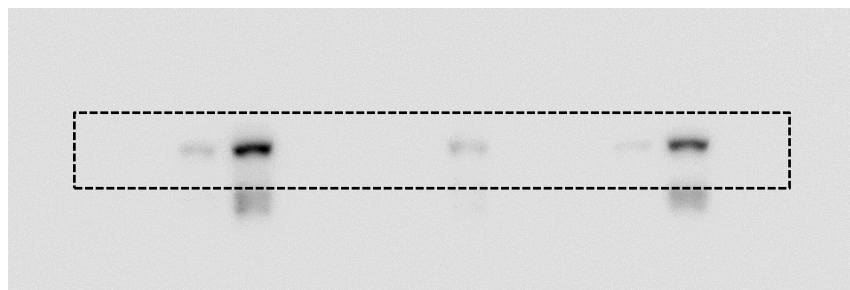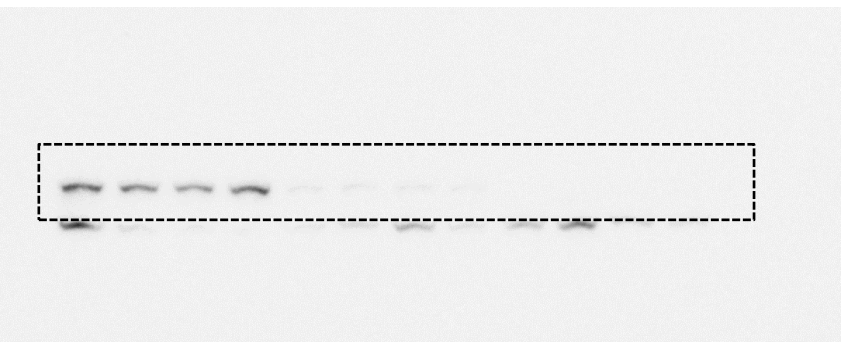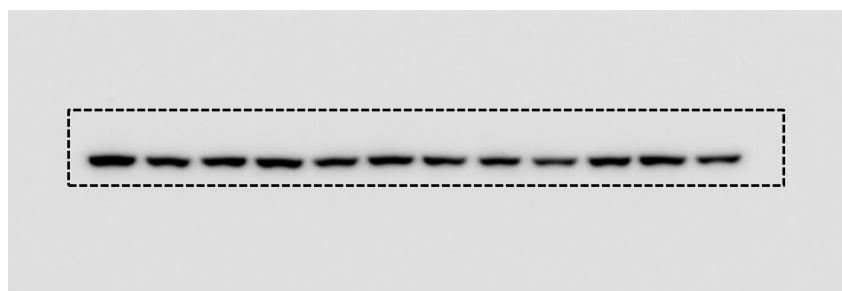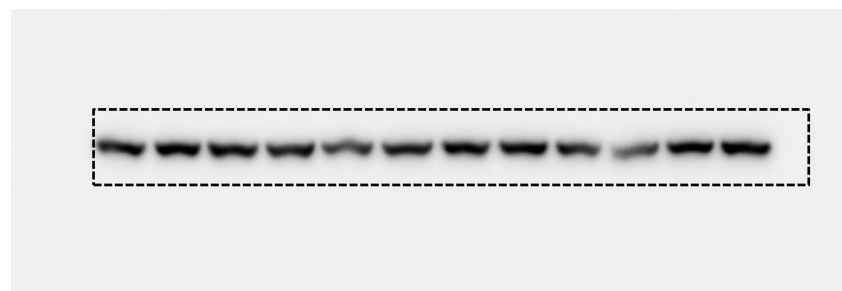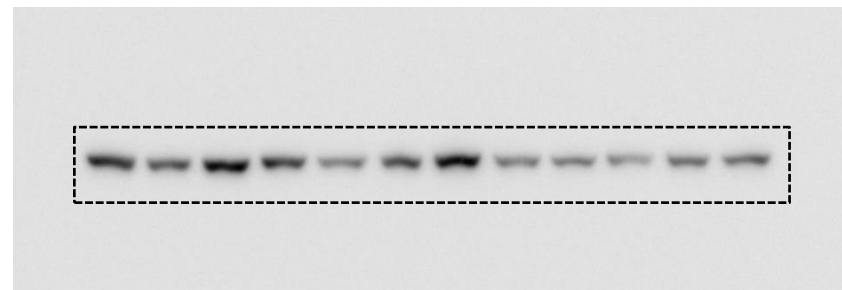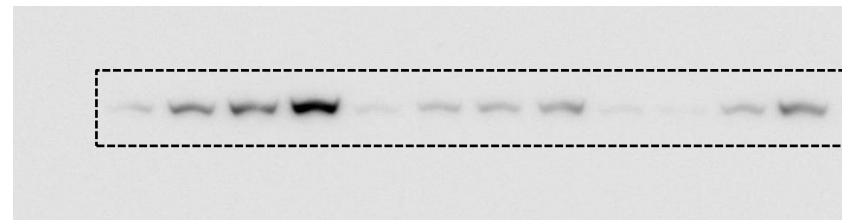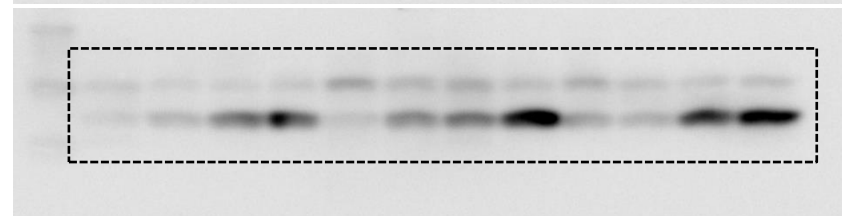

Figure 1A

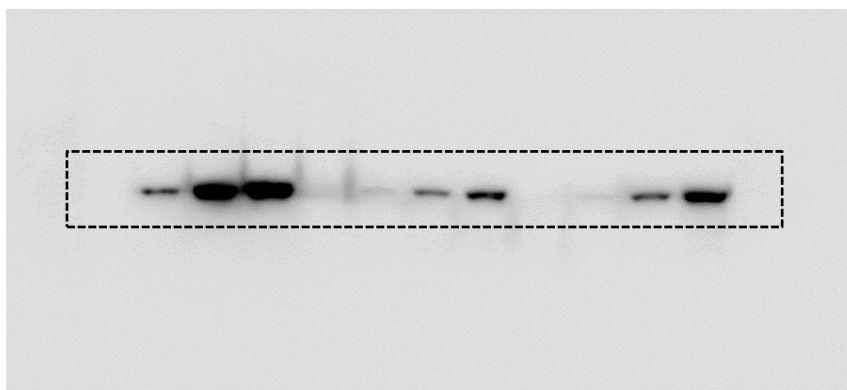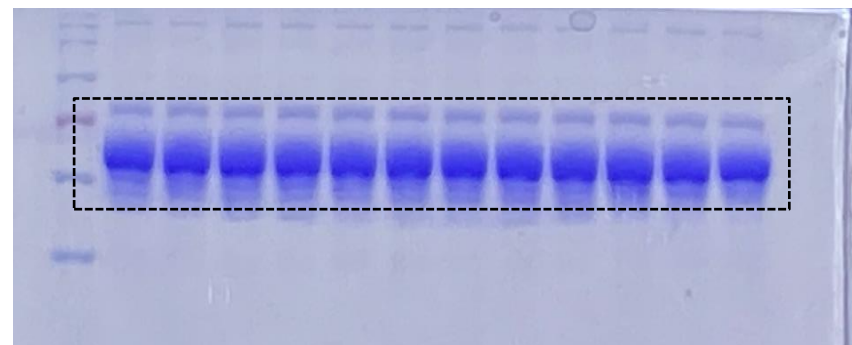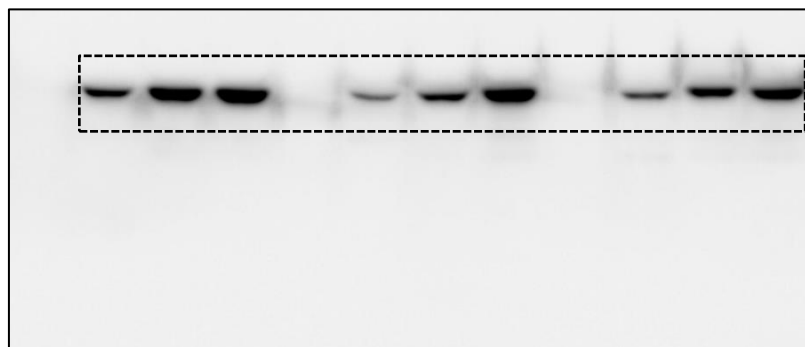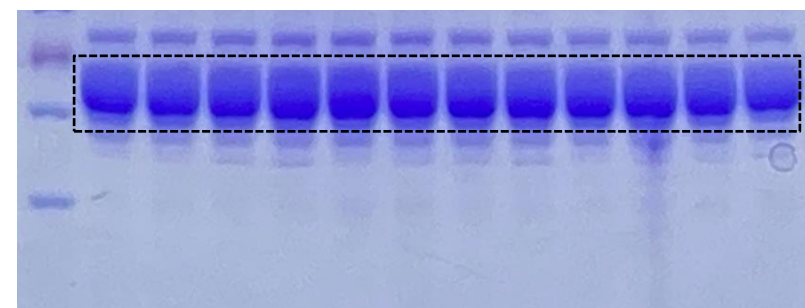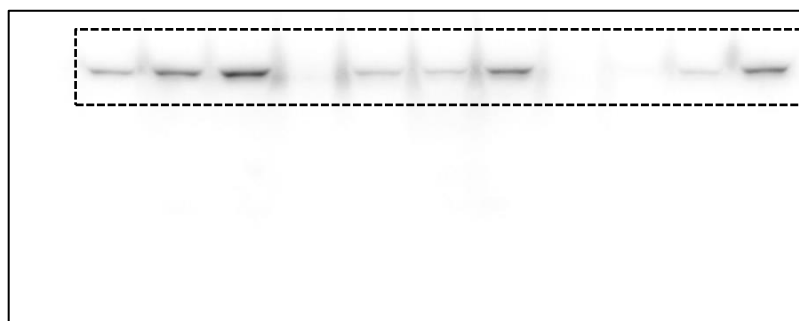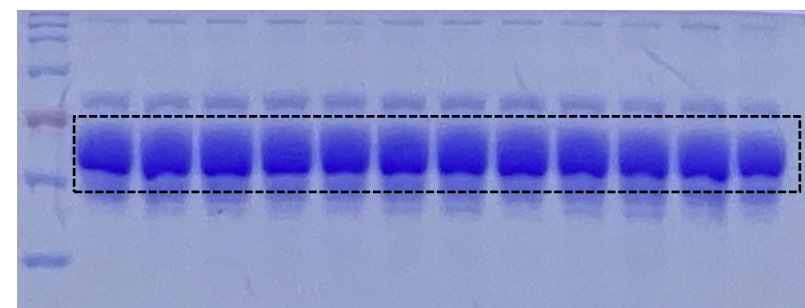

Figure 1B

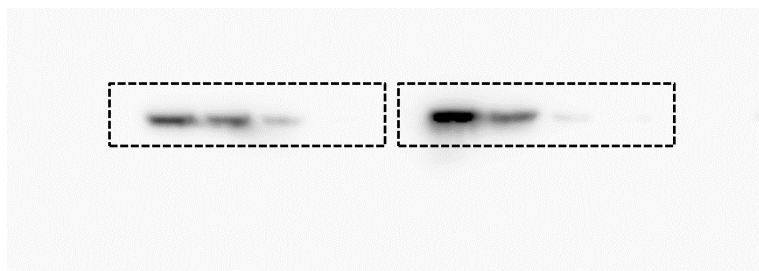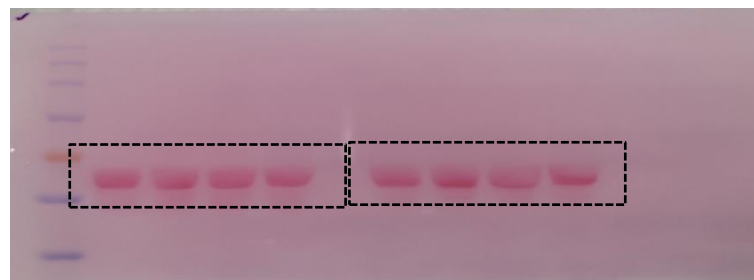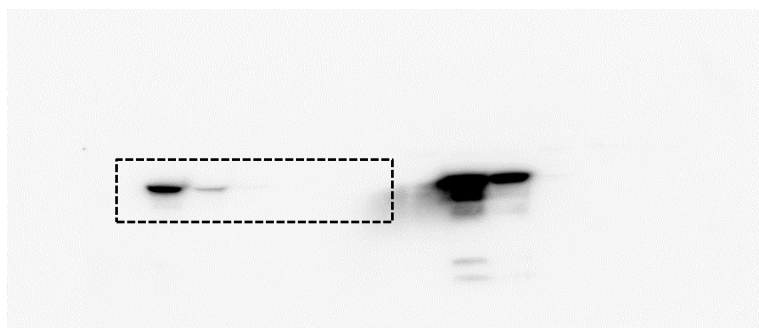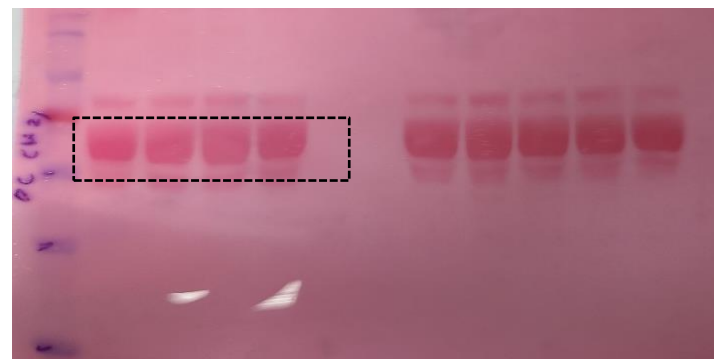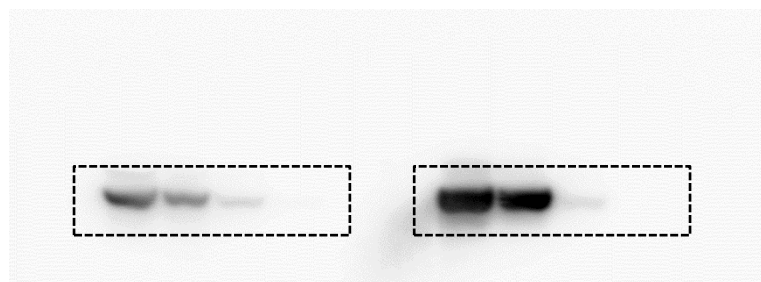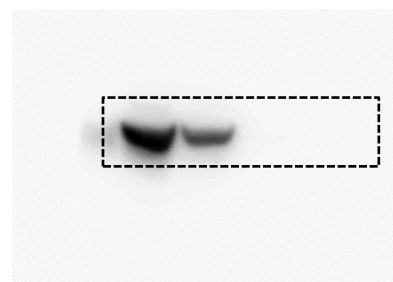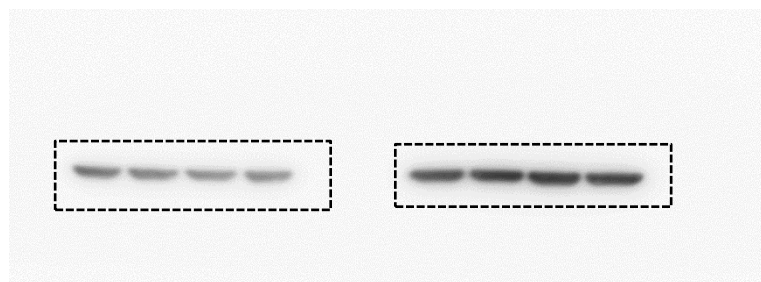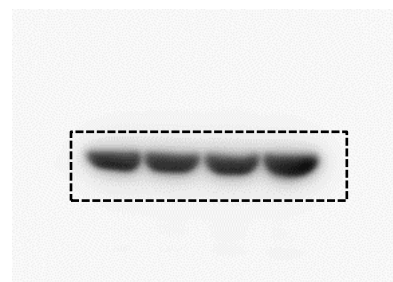

Figure 2A

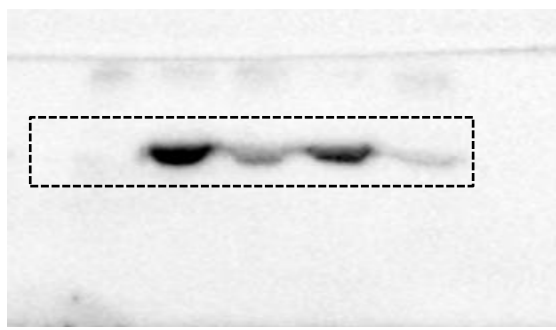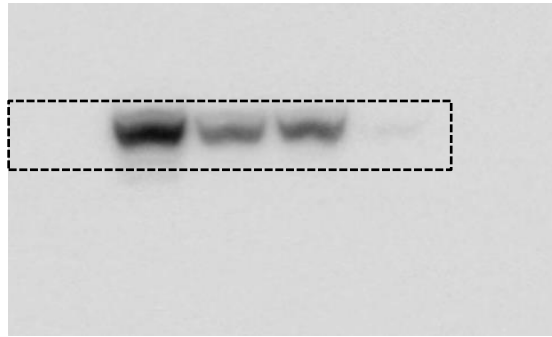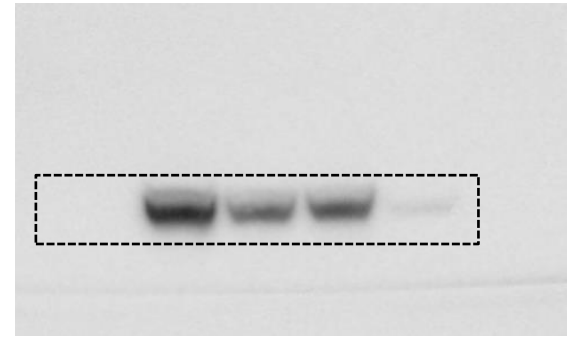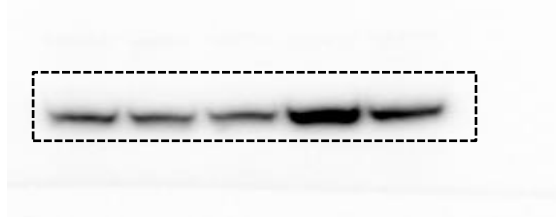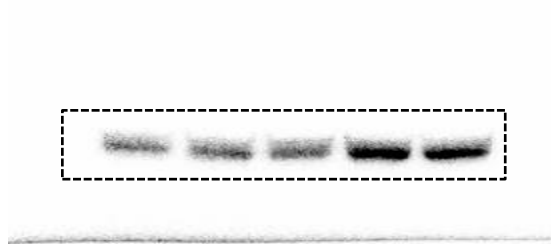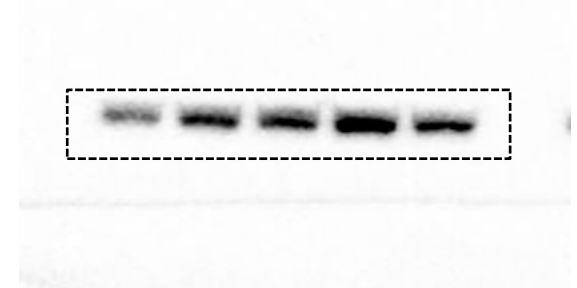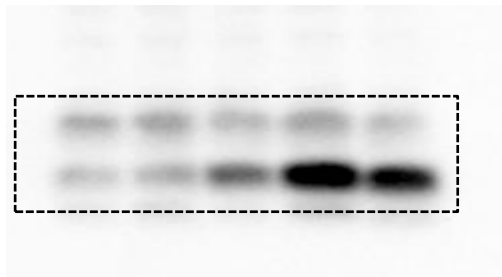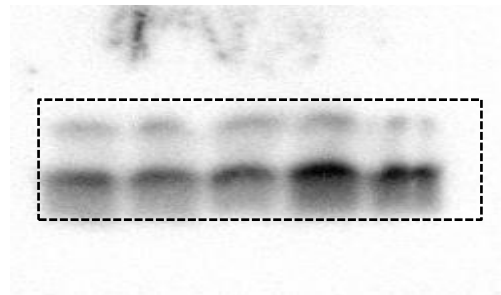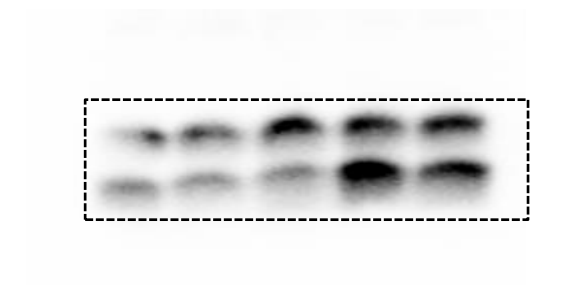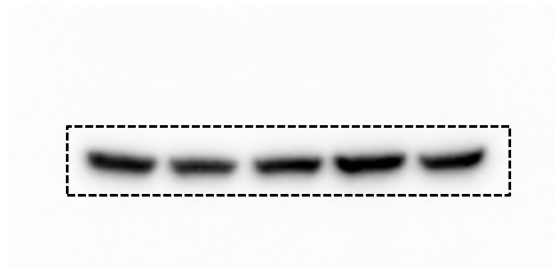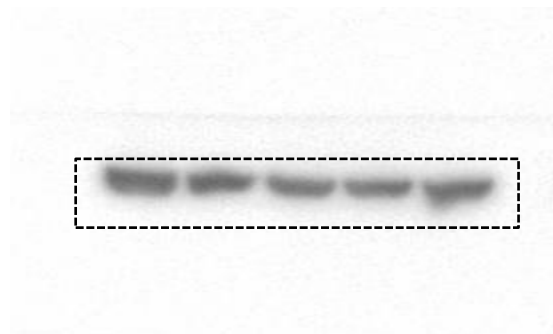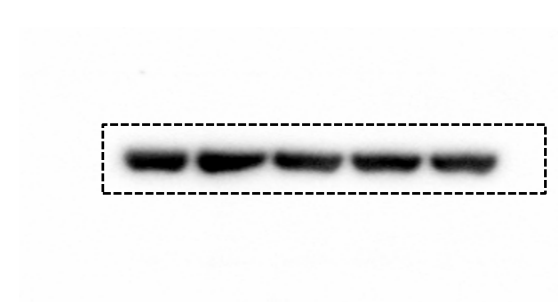

Figure 4A

Supplement: S1 Raw images — (PDF) [file pone.0292309.s001.pdf]
